# Every Breath You Take: Non-invasive Real-Time Oxygen Biosensing in Two- and Three-Dimensional Microfluidic Cell Models

**DOI:** 10.3389/fphys.2018.00815

**Published:** 2018-07-03

**Authors:** Helene Zirath, Mario Rothbauer, Sarah Spitz, Barbara Bachmann, Christian Jordan, Bernhard Müller, Josef Ehgartner, Eleni Priglinger, Severin Mühleder, Heinz Redl, Wolfgang Holnthoner, Michael Harasek, Torsten Mayr, Peter Ertl

**Affiliations:** ^1^Institute of Applied Synthetic Chemistry, Institute of Chemical Technologies and Analytics, Institute of Chemical, Environmental and Bioscience Engineering, Vienna University of Technology, Vienna, Austria; ^2^Austrian Cluster for Tissue Regeneration, Vienna, Austria; ^3^Ludwig Boltzmann Institute for Experimental and Clinical Traumatology, Allgemeine Unfallversicherungsanstalt (AUVA) Research Centre, Vienna, Austria; ^4^Institute of Analytical Chemistry and Food Chemistry, Graz University of Technology, NAWI Graz, Graz, Austria

**Keywords:** microfluidics, 3D culture, biosensor, oxygen, oxygen gradient, organ-on-a-chip, lab-on-a-chip, hydrogel

## Abstract

Knowledge on the availability of dissolved oxygen inside microfluidic cell culture systems is vital for recreating physiological-relevant microenvironments and for providing reliable and reproducible measurement conditions. It is important to highlight that *in vivo* cells experience a diverse range of oxygen tensions depending on the resident tissue type, which can also be recreated *in vitro* using specialized cell culture instruments that regulate external oxygen concentrations. While cell-culture conditions can be readily adjusted using state-of-the-art incubators, the control of physiological-relevant microenvironments within the microfluidic chip, however, requires the integration of oxygen sensors. Although several sensing approaches have been reported to monitor oxygen levels in the presence of cell monolayers, oxygen demands of microfluidic three-dimensional (3D)-cell cultures and spatio-temporal variations of oxygen concentrations inside two-dimensional (2D) and 3D cell culture systems are still largely unknown. To gain a better understanding on available oxygen levels inside organ-on-a-chip systems, we have therefore developed two different microfluidic devices containing embedded sensor arrays to monitor local oxygen levels to investigate (i) oxygen consumption rates of 2D and 3D hydrogel-based cell cultures, (ii) the establishment of oxygen gradients within cell culture chambers, and (iii) influence of microfluidic material (e.g., gas tight vs. gas permeable), surface coatings, cell densities, and medium flow rate on the respiratory activities of four different cell types. We demonstrate how dynamic control of cyclic normoxic-hypoxic cell microenvironments can be readily accomplished using programmable flow profiles employing both gas-impermeable and gas-permeable microfluidic biochips.

## Introduction

With the emergence of advanced cell-based *in vitro* models, which resemble the architecture and physiology of actual native tissue, the ability to control and manipulate cellular microenvironment has become an important aspect in microfluidic cell culture systems. Spatio-temporal control over the cellular microenvironment includes (i) physical forces such as shear stress, (ii) biological cues such as direct and indirect cell–cell interactions, and (iii) chemical signals such as pH, oxygenation, and nutrient supply. Among biochemical signals, oxygen plays a key role in regulating mammalian cell functions in human health and disease. It is also important to note that oxygen concentration varies tremendously throughout the human body ranging from 14% in lungs and vasculature down to 0.5% in less irrigated organs such as cartilage and bone marrow (Jagannathan et al., 2016). Despite the different demand of oxygen in different tissues, routine cell culture is predominantly conducted under atmospheric oxygen tension of 21%. This elevated levels of oxygen exposure of cells is referred to as hyperoxia and can lead to altered cell behavior (Gille and Joenje, 1992). For instance, studies have shown that physiologic oxygen tension modulates stem cell differentiation (Mohyeldin et al., 2010), neurogenesis (Zhang et al., 2011), and is involved in a number of cellular mechanisms needed to maintain tissue function (Pugh and Ratcliffe, 2003; Volkmer et al., 2008). In turn, prolonged oxygen deprivation in a hypoxic oxygen milieu can result in a variety of human pathologies including cancer (Pouysségur et al., 2006), tumor development (Harris, 2002), necrosis (Harrison et al., 2007), infection (Zinkernagel et al., 2007), and stroke (Hossmann, 2006). The importance of monitoring and control of oxygen levels in mammalian cell cultures has therefore led to the implementation of a wide variety of sensing strategies ranging from standard electrochemical electrodes (Nichols and Foster, 1994) and enzymatic sensors (Weltin et al., 2014) to fluorescent and luminescent optical biosensors (Wolfbeis Otto, 2015; Ehgartner et al., 2016b). Of these methods, optical detection based on oxygen-sensitive dyes that are embedded in a polymer matrix are ideally suited for the integration in lab-on-a-chip systems due to the facile integration of sensor spots in microfluidic channels, their long-term stability, reliability, and cost-effectiveness of the sensing probes (Wang and Wolfbeis, 2014; Lasave et al., 2015; Sun et al., 2015). Luminescent intensity as well as decay time of the phosphorescent indicator dye is affected by the amount of the surrounding molecular oxygen, thus providing information on the local oxygen concentration (Gruber et al., 2017). Especially porphyrin-based sensor dyes are well suited for oxygen monitoring in cell-based microfluidic devices due to their high sensitivity, biocompatibility, and reversible quenching behavior (Ungerbock et al., 2013; Ehgartner et al., 2014). Typically, time-resolved optical oxygen monitoring of microfluidic cell culture systems is performed using a measurement set-up consisting of the biochip, optical fibers, a read-out system, and a data acquisition device (Oomen et al., 2016; Gruber et al., 2017). As an example, a polydimethylsiloxane (PDMS) microfluidic chip with oxygen flow-through sensors at the inlet and outlet and an optical oxygen sensor in the cell culture chamber has been realized for a real-time monitoring of respiratory rates of mouse embryonic stem cell cultures and for Chinese hamster ovary cells in monolayers for several days of culture (Super et al., 2016). Oxygen sensor spots were also integrated into microfluidic reaction chambers made of glass and silicon for monitoring of biocatalytic transformations (Ehgartner et al., 2016b). To create a more accessible read-out strategy for a broader research audience, a color CCD-camera mounted to a fluorescent microscope was used for 2D sensing of oxygen distribution inside microfluidic microchannels (Ungerbock et al., 2013). An additional multi-parametric analysis setup was established for simultaneous detection of oxygen and pH using core-shell nanoparticles incorporating oxygen- and pH-sensitive dyes (Ehgartner et al., 2016a). Despite these recent advances, little is still known on spatio-temporal variations of oxygen concentrations inside microfluidic 2D and 3D cell culture systems.

**FIGURE 1:**
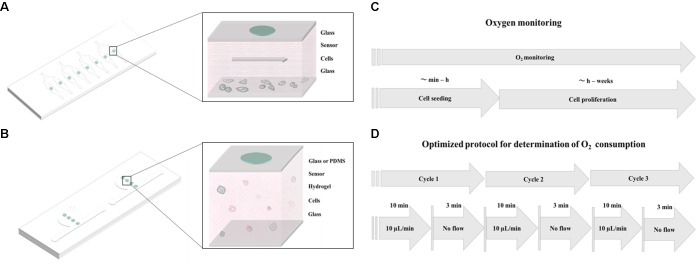
Schematic overview of **(A)** monolayer culture chip and **(B)** 3D cell culture chip with integrated oxygen sensor spots immobilized on the top glass substrate. **(C)** Workflow of oxygen monitoring during cell seeding and cell proliferation studies. **(D)** Workflow of the optimized protocol for evaluation of oxygen consumption of 2D cell cultures.

In this method paper, we investigate the influence of cell numbers, extra-cellular matrix (ECM) surface coatings, different cell types, as well as oxygen permeability of chip materials to determine the key microfluidic parameters needed to reliably monitor and precisely control oxygen levels during 2D and 3D cell cultivation. To gain deeper insight into oxygen supply to and demand of 2D and 3D cell cultures, we have developed two different microfluidic devices containing embedded sensor arrays as shown in **Figures 1A,B**. Material selection in particular influences gas exchange through the bulk chip material, which therefore directly influences the ability to control oxygen within the microfluidic environment. Initially, we have established two protocols for oxygen monitoring; **Figure 1C** shows the general methodology that can be used for oxygen monitoring of cell cultures during seeding, adhesion, and long-term cultivation. Additionally, another protocol, referred to hereafter as “optimized protocol,” is established for precise determination of oxygen consumption rates in 2D microfluidic cultures as shown in **Figure 1D**. Further, we have compared oxygen consumption of cells from epithelial, endothelial, and mesenchymal origin including cancer cells (A549 lung epithelial cells) and primary cells [e.g., normal human dermal fibroblasts (NHDF), adipose-derived stem cells (ASC), and human umbilical vein endothelial cells (HUVEC)]. The effect of a controlled oxygen gradient throughout fibrin hydrogels during vascular network formation over a 7-day culture period is demonstrated using a 3D vascular co-culture model based on HUVEC endothelial cells and ASC stem cells. A final practical application of the oxygen monitoring method involves the early identification of cell necrosis within hydrogel constructs, thus providing quality control information on 3D-cell-laden hydrogel and microtissue cultures.

## Results and Discussion

### Non-invasive Oxygen Monitoring in 2D Microfluidic Cell Cultures

**FIGURE 2:**
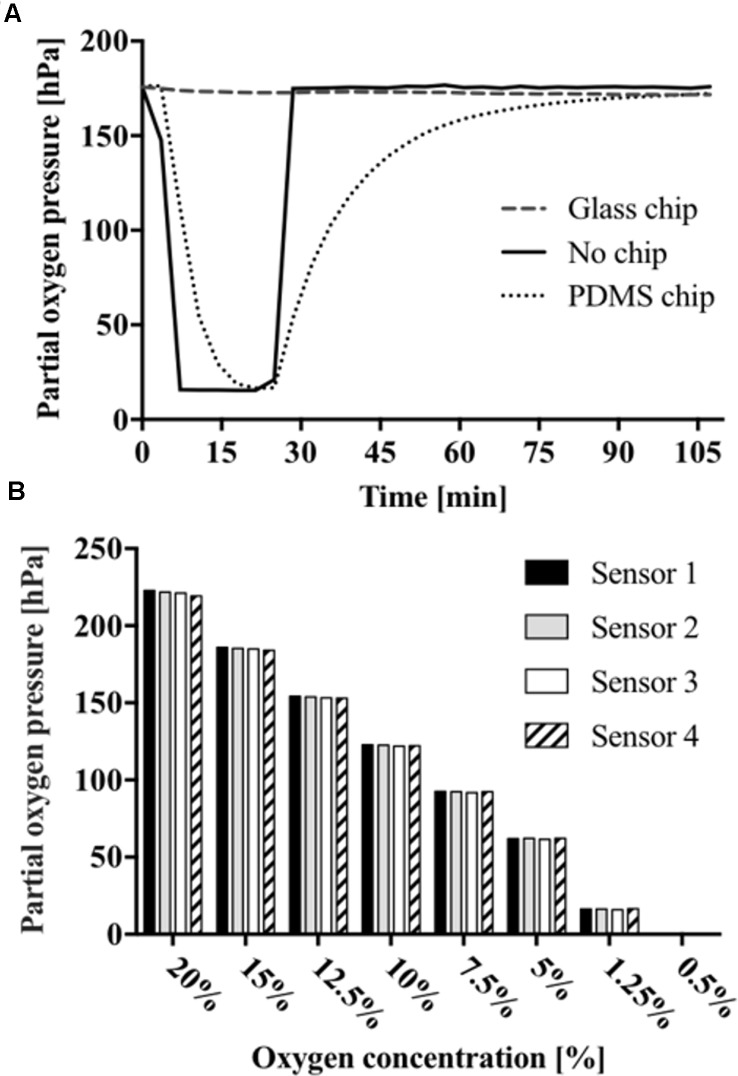
**(A)** Partial oxygen pressures off- and on-chip fabricated from glass or PDMS. **(B)** Measurements presenting sensor-to-sensor variation and sensor response to declining oxygen concentration starting from 0.5 to 20%.

Prior to oxygen monitoring of 2D cell cultures, the influence of chip material permeability was evaluated based on the response of the oxygen-sensitive microparticle-based sensors. Therefore, gaseous oxygen content within sealed microchannels was monitored for microfluidic chips fabricated either from oxygen-permeable PDMS or impermeable glass (see **Supplementary Figure S1**). Both devices were simultaneously exposed to an oxygen environment of 2.5% for 20 min before re-oxygenation. As shown in **Figure 2A**, no sensor response was observable for gas-impermeable glass chips with no detectable decrease in partial oxygen pressure. Microfluidic chips made of gas-permeable PDMS showed a decrease in partial oxygen pressure from 176.4 to 15.4 hPa with a *t*_90_-value of 15 min and a delay in full recovery to atmospheric oxygen after 87 min. These results demonstrate the importance of material selection during microfluidic chip fabrication. Differences in de- and re-oxygenation linked to material permeability offer the opportunity to either inhibit or allow external oxygen supply to the cell cultures. In turn, sensor-to-sensor variation was evaluated using oxygen concentrations ranging from 20 to 0.5% via small gas feeding by a CO_2_/O_2_ controller equipped with zirconium-based oxygen sensor, which was connected to the inlet ports of the microfluidic chips. As shown in **Figure 2B**, sensor-to-sensor variation among the individual sensor spots was 221.2 ± 1.3 hPa for 20%, 122.4 ± 0.3 for 10%, 61.9 ± 0.2 hPa for 5%, and 0.2 ± 0.1 hPa for 0.5% oxygen, respectively. These results show excellent reproducibility despite the manual dispensing of the sensor spots into the microfluidic devices.

**FIGURE 3:**
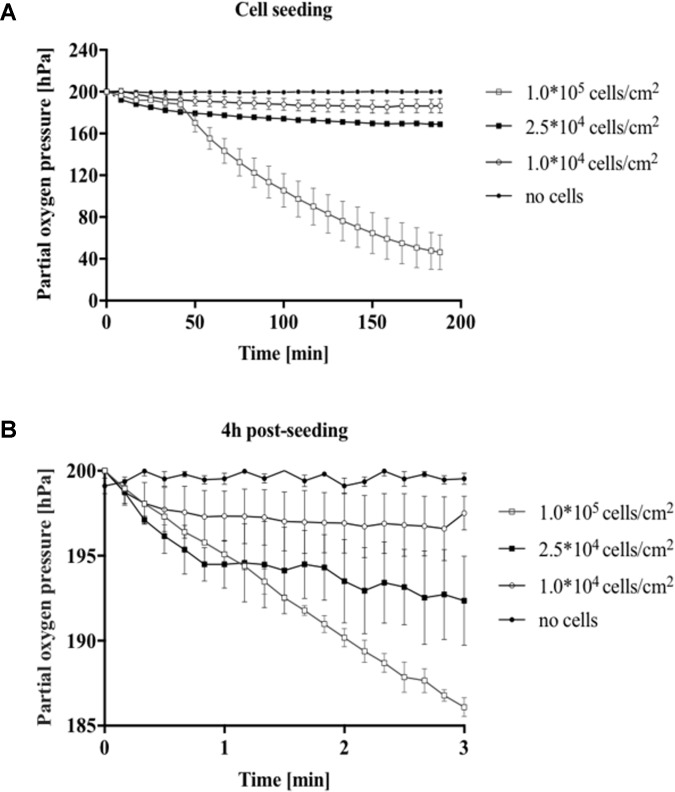
Impact of cell density for A549 cells on oxygen consumption during cell seeding and after cell adhesion. **(A)** Partial oxygen pressure recorded during 3 h of cell seeding and **(B)** oxygen consumption 4 h post-seeding using the optimized oxygen consumption protocol.

In the next set of experiments, the impact of cell density on oxygen consumption was evaluated during cell seeding and for fully attached cell cultures. A549 lung epithelial cells were seeded in gas-impermeable microfluidic chips at initial cell seeding densities ranging from 1 × 10^4^ to 1 × 10^5^ cells/cm^2^. **Figure 3A** shows a partial oxygen pressure around 200 hPa right after seeding, which accurately represents ambient oxygen levels in cell culture media at 37°C. Difference in partial oxygen pressure for increasing cell numbers was observable already 10 min after cell seeding, thus pointing at higher oxygen demand in the presence of larger cell numbers. Interestingly, while similar oxygen depletion values were observed in the first 40 min, rapid increase in oxygen consumption was found already after 50 min for 1.0 × 10^5^ cells/cm^2^ (see **Supplementary Figure S2**), which can be linked to completed cell adhesion and start of exponential cell growth (Rehberg et al., 2013). Cell densities of 1 × 10^4^ and 2.5 × 10^4^ cells/cm^2^ resulted in moderate total oxygen depletion values of 13.4 ± 6.7 hPa and 31.1 ± 1.5 hPa, respectively. A more pronounced oxygen depletion was found for the highest cell density of 1 × 10^5^ cells/cm^2^ of 153.8 ± 16.7 hPa. Results of this study show that in the presence of high cell densities oxygen depletion occurs 3 h after cell seeding when using gas-impermeable glass microfluidic chips. Next, an optimized protocol was established for precise measurements of oxygen consumption rates (see **Figure 1D** for details). To ensure oxygen consumption measurements, a flow rate of 10 μL/min over period of 10 min prior to the measurements was introduced to maintain oxygenated conditions of around 200 hPa. Flow rates below this threshold did not show full oxygenation (data not shown). For the actual determination of oxygen consumption rates, flow was halted and the decrease in oxygen partial pressure was immediately recorded for 3 min in all chambers. **Figure 3B** shows decreasing oxygen levels over a period of 3 min (*n* = 3) in the presence of increasing numbers of fully attached A549 lung cells after 4 h of post-seeding, indicating that sufficient re-oxygenation prior to measurement markedly improves data quality. Calculated total oxygen consumption rates of 0.8 hPa/min for 1 × 10^4^ cells/cm^2^, 2.6 hPa/min for 2.5 × 10^4^ cells/cm^2^, and 4.6 hPa/min for 1 × 10^5^cells/cm^2^. Overall, integration of sensor spots above the cell monolayer culture are sensitive enough to estimate oxygen availability and cell population oxygen demands in microfluidic cell cultivation chambers.

### Impact of Cell Type on Oxygen Consumption Rates

**FIGURE 4:**
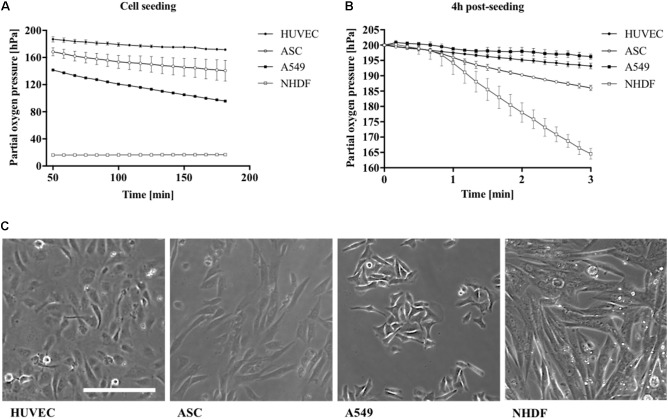
Influence of cell type on partial oxygen pressure during **(A)** cell seeding and **(B)** 4 h post-seeding using the optimized oxygen consumption protocol (*n* = 6). **(C)** Cell morphology of different cell types seeded with same cell numbers per cm^2^. Scale bar, 200 μm.

Differences in oxygen consumption rates between well-established primary cell types (Manning et al., 2015; Haase and Kamm, 2017; Zhang et al., 2017) and cancer cell lines (Cooper et al., 2016) were investigated to further evaluate the presented non-invasive microfluidic oxygen monitoring method. Partial oxygen pressure was monitored during cell seeding for cells from epithelial (A549), endothelial (HUVEC), and mesenchymal origin (NHDF and ASC) at the same initial seeding density of 2.5 × 10^4^ cells/cm^2^ corresponding to 5.5 × 10^3^ cells per microchannel. As shown in **Figure 4A** the different cell types can be readily distinguished by cell type–specific variations in oxygen depletion during adhesion to the surface of the microfluidic channels. NHDF fibroblast cells showed highest oxygen depletion values leveling off at a partial oxygen pressure of 17 hPa, which corresponds to a total oxygen consumption of 183 ± 0.4 hPa oxygen in 3 h during cell adhesion. The oxygen consumption for the other cell types were lower with 104.2 hPa (*n* = 1) for A549 lung cells, 28.4 ± 0.4 hPa for HUVEC endothelial cells, and 59.4 ± 15.4 hPa for ASC stem cells, respectively. Next, oxygen consumption rates were determined using the optimized protocol as shown in **Figure 4B**. Oxygen consumption varied heavily between the different cell types exhibiting low cellular respiratory activity of 3.7 ± 1.2 hPa (1.2 ± 0.4 hPa/min) for A549 lung cells and 6.8 ± 2.1 hPa (2.3 ± 0.7 hPa/min) for HUVEC, 13.9 ± 1.6 hPa (4.7 ± 0.5 hPa/min) for ASC, and highest rates observable of 35.4 ± 2.5 hPa (11.8 ± 0.8 hPa/min) for fibroblast cells. Overall, these results correspond well with literature values the specific cell types demonstrating the good performance of the presented microfluidic oxygen sensing method (Heidemann et al., 1998; Powers et al., 2008; Abaci et al., 2010; Zhang et al., 2014). In addition, **Figure 4C** shows differences in both cell morphology and cell size with a cell size of 72.4 μm^2^ for HUVEC endothelial cells, 71.6 μm^2^ for A549, 232.9 μm^2^ for NHDF fibroblast cells, and 125.3 μm^2^ for ASC stem cells. Cell size directly correlates with oxygen consumption, (Wagner et al., 2011) which explains the high respiratory activity of fibroblast cells displaying the biggest cell size.

### Impact of Cell Adhesion Promoters on Oxygen Consumption Rates

**FIGURE 5:**
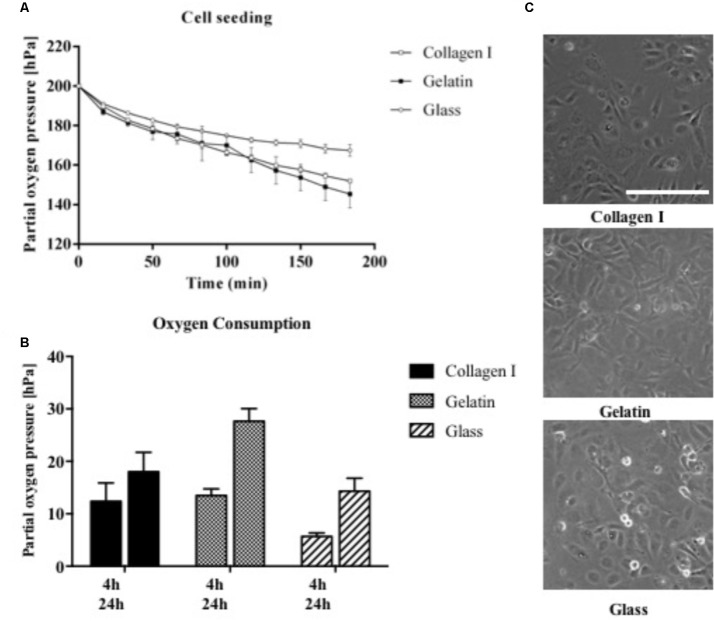
**(A)** Influence of cell adhesion promoters on partial oxygen pressure during cell seeding for surfaces treated with collagen I and gelatin in comparison to untreated glass (*n* = 6). **(B)** Oxygen consumption rates of HUVEC endothelial cells on treated and untreated glass surfaces recorded after 4 and 24 h using the optimized oxygen consumption protocol (*n* = 6). **(C)** Morphology of HUVEC endothelial cells on treated and untreated glass surfaces 4 h post-seeding. Scale bar, 200 μm.

Since mammalian cells strongly interact and respond to surface properties such as the biocompatibility and functionalization of the interface, the influence of ECM surface promoters including 1% gelatin and collagen I was investigated on the respiratory activity of HUVEC endothelial cells during cell seeding and 3 h after attachment. As shown in **Figure 5A** already 3 min following the introduction of endothelial cells seeded on gelatin and collagen I-coated microchannels, a drop in partial oxygen pressure by 1 hPa was recorded. In contrast, partial oxygen pressure of 200 hPa remained in the absence of cells. In turn, after completion of cell attachment and establishment of endothelial cell monolayers, both adhesion promoters initiated elevated respiratory activities in the range of 130–150 hPa compared to untreated glass with partial oxygen pressure around 165.7 ± 5 hPa. **Figure 5B** shows that respiration of HUVEC endothelial cells further increased from 4 to 24 h post-seeding by 140% for collagen I coating and 170% for gelatin-coated microchannels. Even though respiratory activity of HUVEC endothelial cells cultured on untreated glass displayed highest relative increase of 250% after 24 h, however, absolute respiration rates were 20–40% lower than cells cultivated on ECM-like adhesion promoters. These findings can be explained by incomplete adhesion of cells on untreated surfaces, where 10% of the cells were only partially attached as shown in **Figure 5C**. In other words, our results demonstrate that our non-invasive on-chip oxygen detection method can also be applied for adhesion and biocompatibility studies, where metabolic assays as well as microscopic inspection of cell phenotype alone can result in overestimation of cellular activity.

### Non-invasive Oxygen Monitoring in Three-Dimensional Microfluidic Cell Cultures

**FIGURE 6:**
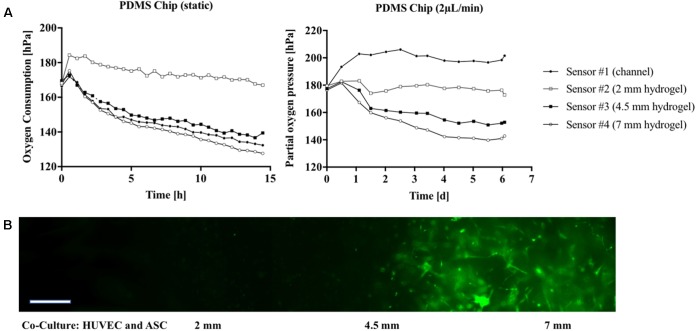
**(A)** Partial oxygen pressure during cultivation of vascular co-cultures in gas-permeable PDMS microfluidic recorded 14 h post-seeding (no flow conditions; Left) followed by a dynamic cultivation period of 6 days (flow rate 2 μL/min; Right). **(B)** Morphology of the vascular network in glass chips in the presence of flow at 5 μL/min at day 6 post-seeding. GFP-HUVEC endothelial cells are displayed green. Scale bar, 50 μm.

**FIGURE 7:**
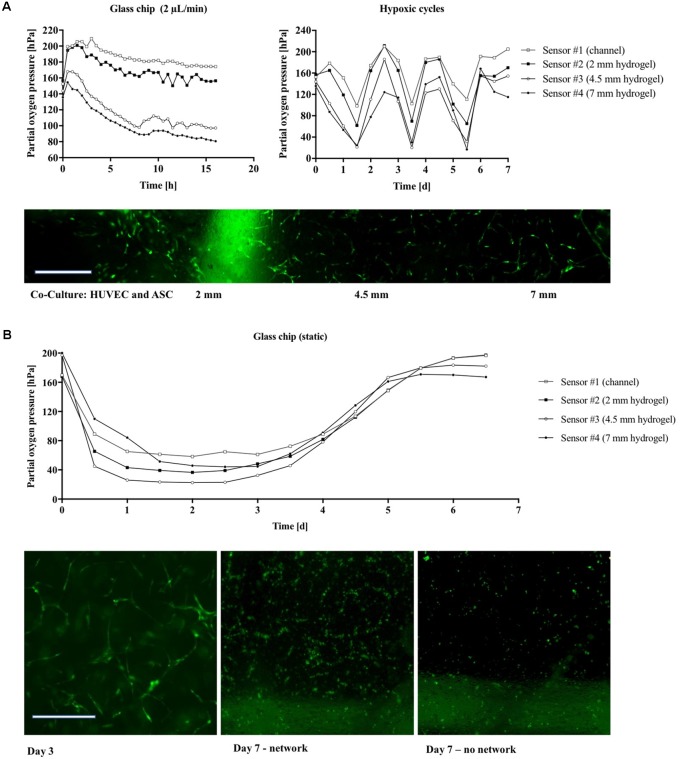
**(A)** Partial oxygen pressure during cultivation of vascular co-cultures in gas-impermeable glass device recorded during the first 16 h (Left) and during long-term cultivation for 6 days (Right). **(B)** Morphology of the vascular network in glass chips in the absence of nutrient and oxygen supply. GFP-HUVEC endothelial cells are displayed green. Scale bar, 50 μm.

Since state-of-the-art *in vitro* models frequently employ 3D-hydrogel-based cell cultures, the integrated oxygen sensor array was evaluated using a well-established co-culture model of ASC stem cells with HUVEC endothelial cells (Muhleder et al., 2018). Here, an oxygen-permeable PDMS-based microfluidic biochip containing four spatially resolved sensor spots (see also **Figure 1B**) was seeded with a HUVEC/ASC cell co-culture. Oxygen monitoring was performed for medium supply channels (sensor #1) as well as at 2 to 7 mm within the fibrin hydrogel (sensors #2–4). On the first day of culture, cell co-cultures settled and established direct cell-cell contacts and reciprocal exchange of pro-angiogenic factors using a static stop-flow cultivation period over 24 h. **Figure 6A** (left panel) shows that after 45 min of hydrogel polymerization, partial oxygen pressure of the feeding channel stayed constant around 169.0 ± 1.1 hPa for the first day of culture. In contrast, within the hydrogel construct oxygen levels slowly decreased over time to around 132.4 ± 5.4 hPa independent of diffusion distance through the hydrogel with an average oxygen decrease of 2.5 hPa/h. After the first day of culture, continuous medium supply was initiated at a flow rate of 2 μL/min for the next 5 days of culture. **Figure 6A** (right panel) shows that at day 6 of vascular network formation, an oxygen gradient was established starting at steady atmospheric partial oxygen pressure of around 198.4 hPa for sensor #1 at the medium feeding channel, and linear decreasing values of 176.3 hPa for sensor #2 at 2 mm, 152.1 hPa for sensor #3 at 4.5 mm, and 140.9 hPa for sensor #4 at 7 mm distance from the feeding channel. Overall, average decrease of partial oxygen pressure of 10 hPa per day was observed for the deepest regions of the hydrogel construct. **Figure 6B** shows that even though a linear oxygen gradient was established, vascular network formation with interconnecting endothelial tubes started at a distance of 4.5 mm from the feeding channel. This means that a reduction of partial oxygen pressure by 70 hPa does not affect vascular network formation. In turn, similar vascularization experiments were performed for oxygen-impermeable glass-based microfluidics to generate stronger oxygen gradients. Due to limited oxygen supply from the humidified incubator atmosphere through the bulk chip material, oxygen can exclusively be controlled by adjustment of medium flow rate, thus amount of dissolved oxygen fed to the HUVEC/ASC cell co-culture. To elucidate this mechanism, *in silico* experiments performed using finite volume CFD simulations (Ansys FLUENT^®^ 6.3.26) of oxygen distribution in fibrin hydrogel in the absence of cellular respiratory load were conducted to determine the oxygen saturation within the fibrin hydrogel by medium supply exclusively. **Supplementary Figure S3** shows that already during the first 60 min of medium perfusion, 50% of the supplied oxygen diffuses 2 mm into the hydrogel chamber. Rapid oxygen diffusion then results in a supply of 75% of dissolved oxygen into the depth of the chamber after 6 h and complete oxygen saturation of the chamber within 12 h after initiation of perfusion with the theoretical assumption of oxygen-free conditions at time point zero. **Figure 7A** (left panel) shows that during perfused hydrogel culture within oxygen-impermeable microfluidics right after 45 min of hydrogel polymerization, at a flow rate of 2 μL/min oxygen saturation at a partial oxygen pressure of 197.1 ± 3.2 hPa was observable for the medium feeding channel as well as up to 2 mm into the hydrogel construct. At distances between 4.5 and 7 mm, partial oxygen pressures decreased to 161.2 ± 9.5 hPa with a mean oxygen depletion rate of 5 hPa/h, which is twofold higher compared to the rate found with oxygen-permeable microfluidic chips. In turn, **Figure 7A** (right panel) shows that the combination of impermeable microfluidic biochips with optical in-line oxygen monitoring can be used to control oxygen within microfluidic cultures. By exploiting the respiratory activity of cells within the 3D hydrogel construct, this method can be used to control oxygen levels during long-term cultivation of 3D cells cultures without the need for external oxygen controllers such as CO_2_/O_2_ incubators, gas controls units for gas mixing, as well as nitrogen gas- or oxygen-scavenging chemicals for oxygen depletion. A pre-programmed flow profile in the range of 0–2 μL/min was used to expose cells to alternating cycles of normoxic and hypoxic culture conditions with peak-to-peak values for partial oxygen pressure of max. 200 hPa down to min. 17 hPa and an average cycle time around 2.5 h over several days for 3D cell culture. **Figure 7A** (bottom panel) shows that due to the cycling of normoxic and hypoxic conditions, formation of a vascular network with decreased length and thickness of vascular sprouts was observable; however, more even distribution of the network was observable starting already at 2 mm distance from the medium feeding channel. In a final set of experiments, we demonstrated how the presented non-invasive microfluidic oxygen monitoring method can be applied not only for oxygen control, but also for evaluation of cell health status during oxygen- and nutrient-dependent limitation of three-dimensional cultures in the absence of medium perfusion. As shown in **Figure 7B** (top panel) an oxygen gradient was established in the absence of medium perfusion due to the respiratory activity of intact cell co-cultures until day 3 post-seeding corresponding to vascular network formation (see **Figure 7B** bottom left image). Over the course of 36 h of nutrient and oxygen limitation, partial oxygen pressure gradually recovered to fully oxygenated conditions around 200 hPa, which is the result of cell death by nutrient and oxygen depletion over 6 days. **Figure 7B** shows a residual pattern of GFP-containing cell remnants (visible in the bottom center image) that resembles the vascular network morphology from day 3. These patterns were not observable for cell co-cultures closest to the medium feeding channel, which did not form vascular networks (bottom right image).

## Conclusion

In this method paper, we demonstrate the ability to reliably and reproducibly detect dissolved oxygen levels and cellular oxygen demands in 2D and 3D microfluidic cell culture systems. The integration of oxygen-sensitive microparticle-based sensor spots enables time-resolved monitoring of partial oxygen pressures, thus allowing the estimation of cellular oxygen consumption rates of cell monolayers in the presence of increasing flow rates, varying cell numbers, cell types, and ECM-coatings. The total oxygen consumption observed for the different cell densities increased linearly. This means that the presented measurement method has the potential to be used for indirect monitoring of cell viability in toxicological screening studies. Additionally, we show that metabolic evaluation of complex 3D tissue-engineered constructs can be accomplished using biologically relevant co-cultures. The ability to monitor complex 3D physiological cellular microenvironments further highlights the benefits of sensor integration in microfluidic systems to control, mitigate, and accurately predict cell behavior (Charwat et al., 2013, 2014; Ertl et al., 2014; Mahto et al., 2015; Rothbauer et al., 2015a,b). The presented on-chip-integrated oxygen-sensing method is well suited for applications in advanced organ-on-a-chip systems, because it enables non-invasive, real-time, label-free, *in situ* monitoring of oxygen demands, metabolic activity, oxygen uptake rates, and cell viability. We believe that the integration of cost-efficient and reliable sensor technology in microfluidic devices holds great promise for the reliable establishment of physiologic or pathologic tissue conditions, thus opening new avenues in biomedical research and pharmaceutical development.

## Materials and Methods

### Preparation of the Oxygen Sensor

Amine-functionalized polystyrene beads (500 μL of a 50 mg/mL stock solution, micromer^®^ product code 01-01-303, micromod, Germany) were diluted with water (2 mL) and THF (200 μL). This dispersion was stirred for 30 min at room temperature. Afterward, an oxygen indicator solution [188 μL of a THF solution containing 2 mg/mL platinum (II) meso-tetra (4-fluorophenyl) tetrabenzoporphyrin (PtTPTBPF)] was added dropwise, the dispersion was sonicated for 30 min, centrifuged at a relative centrifugation force of 6200, and the supernatant was decanted. The particles were purified using multiple washing, centrifugation, and decantation steps. The purification process was repeated with water (1–2 times) at the beginning, followed by ethanol (3–5 times) and finished with water (1–2 times). After the final decantation step the particles were diluted with water to a particle concentration of approx. 50 mg/mL. Characterization and calibration of oxygen sensor was performed as described previously (Ehgartner et al., 2016b).

### Microfluidic Chips and Sensor Integration

The microdevices for monolayer cultures were comprised of two glass substrates (VWR) bonded together with adhesive film containing the fluidic structure. Microfluidic chambers and channels were designed with CAD (AutoCAD 2017) and cut into the adhesive film (ARcare 8259 and ARseal 90880, Adhesive Research, Ireland) with a desktop vinyl cutter (GS-24 Desktop Cutter, Roland DGA Corporation, Germany). Three layers of adhesive film were used to obtain a chamber height of 460 μm. The chip contained 8 cell-culture chambers of 0.22 cm^2^ (10 μL volume) with one inlet connected to two chambers with a 400 μm wide channel (**Figure 1A**). Holes for inlets and outlets were drilled into the upper glass substrate before integration of sensor particles. Oxygen sensor microparticles were applied by pipetting directly to the glass substrate. After drying for 2 h at room temperature, the microparticles were immobilized to the glass substrate and the fluidic structures were sealed with adhesive film via pressure activation. Devices for three-dimensional (3D) hydrogel cultures had a circular hydrogel chamber (65 μL volume) adjacent to a medium channel (see **Figure 1B**). Microstructures were either fabricated by soft lithography using PDMS (Sylgard^®^ 184 Silicone Elastomer Kit, Down Corning, Germany) from 3D-printed molds (immaterialize, Denmark) or sandblasted into glass slides. Oxygen-sensitive conjugated microparticles were again applied via pipetting directly to the glass substrate and immobilized after 2 h of drying. PDMS devices were sealed by plasma bonding to glass slides using air plasma (Harrick Plasma, High Power, 2 min) and glass devices were sealed using adhesive film (ARseal 90880, Adhesive Research, Ireland).

### Cell Culture

A549 human lung carcinoma epithelial-like cell line (ATCC) were cultured in RPMI 1640 Medium (Gibco) supplemented with 10% fetal bovine serum, L-glutamine, and 1% antibiotic/antimycotic solution. NHDF (Lonza) were cultured in high glucose Dulbecco’s modified Eagle’s medium (DMEM, Sigma Aldrich) supplemented with 10% fetal bovine serum, 2 mM glutamine, and 1% antibiotic-antimycotic solution. Primary human ASC and HUVEC were isolated as previously described (Petzelbauer et al., 1993; Wolbank et al., 2007; Priglinger et al., 2017) and maintained in fully supplemented endothelial cell growth medium 2 (EGM-2, PromoCell) with 5% fetal calf serum until passage 5–9. GFP-labeled endothelial cells were prepared as previously described (Knezevic et al., 2017; Muhleder et al., 2018) All cell cultures were maintained in a humidified atmosphere at 37°C and 5% CO_2_. Cell culture media used during perfusion of the microfluidic monolayer chambers was supplemented with 0.5% HEPES (Sigma Aldrich). Prior to cell seeding, microfluidic tubing and valves were sterilized with 70% ethanol and rinsed thoroughly with PBS (Sigma Aldrich) and cell culture medium. The assembled microfluidic biochips were disinfected using 70% ethanol and rinsed with sterile PBS prior to coating with 5% collagen I solution (Sigma Aldrich, Austria).

### On-Chip Oxygen Monitoring

**FIGURE 8:**
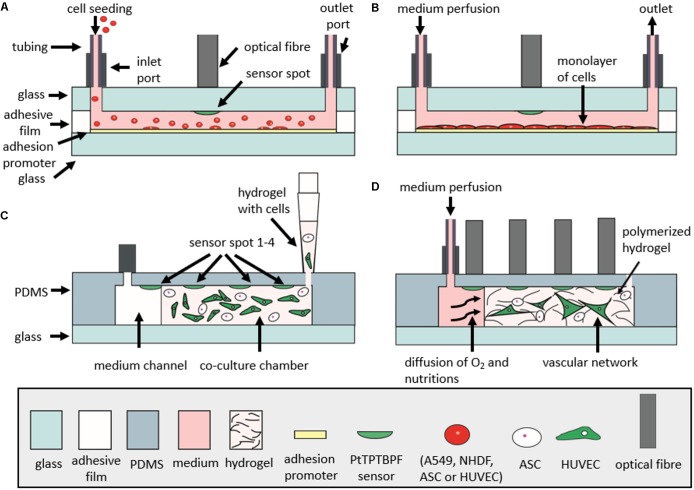
Schematic illustration of microfluidic chips in cross section during cell seeding and cell culturing. **(A)** Monitoring of oxygen consumption during seeding of cells in microfluidic monolayer culture chip. **(B)** Measurement of oxygen consumption rates during the optimized protocol. **(C)** Seeding of cells and hydrogel into gas-permeable 3D cell culture chip. **(D)** Monitoring of oxygen consumption during vascular network formation in 3D cell culture chip with a continuous perfusion of medium. For the gas-impermeable 3D cell culture chip, the upper layer is made of glass and is bonded to the lower glass substrate with adhesive film (not shown in figure).

Oxygen monitoring was carried out at a sampling frequency of 1 Hz using a FireStingO2 optical oxygen meter (Pyroscience, Germany^1^) connected to optical fibers (length 1 m, outer diameter 2.2 mm, fiber diameter 1 mm). Integrated sensors were calibrated using a CO_2_/O_2_ oxygen controller (CO_2_-O_2_-Controller 2000, Pecon GmbH, Germany) equipped with integrated zirconium oxide oxygen sensors. Oxygen measurements were initiated directly after injection of cell solutions and partial oxygen pressure was monitored up to a maximum duration of 7 days. To evaluate the influence of cell number on oxygen consumption, A549 lung cells were seeded at concentrations of 1 × 10^4^ cells/cm^2^ (2.2 × 10^3^ cells/chamber), 2.5 × 10^4^ cells/cm^2^ (5.5 × 10^3^ cells/chamber), and 1.0 × 10^5^ cells/cm^2^ (22 × 10^3^ cells/chamber). For oxygen monitoring of different cell types, A549 lung epithelial cells, NHDF fibroblast cells, HUVEC endothelial cells, and ASC stem cells were seeded at a concentration of 2.5 × 10^4^ cells/cm^2^ (5.5 × 10^3^ cells/chamber) To investigate the influence of adhesion promoters on oxygen consumption, HUVEC endothelial cells were seeded at a concentration of 2.5 × 10^4^ HUVEC/cm^2^ (5.5 × 10^3^ cells/chamber) on different surfaces including untreated glass 5% collagen I and 1% gelatin. For oxygen monitoring of 3D fibrin hydrogels, ASC stem cells and HUVEC endothelial cells were mixed at a cell density of 5 × 10^5^ cells/mL (32.5 × 10^3^ cells/chamber, 65 μL chamber volume) per cell type in fibrinogen and thrombin (TISSEEL^®^, Baxter, Austria) to obtain a final concentration of 2.5 mg/mL fibrinogen and 1 U/mL thrombin. The hydrogel was loaded into the hydrogel chambers of the microfluidic chip and allowed to polymerize for 45 min before the addition of fully supplemented EGM-2 culture medium. Thereafter, vascular 3D cell cultures were maintained at a flow rate of 2 μL/min. In **Figures 8A–D**, schematic illustrations depict the cell seeding and measurement setup of monolayer culture chips (A–B) and 3D culture chips (C–D).

### Optimized Protocol for On-Chip Oxygen Consumption Measurements

For determination of oxygen consumption, an optimized protocol was established recording three consecutive measuring cycles. Each cycle comprised of an oxygen saturation phase of 10 μL/min for 10 min followed by a recording phase under stop-flow conditions for 3 min.

### CFD Simulation of Oxygen

Oxygen saturation was simulated using a multipurpose finite volume CFD simulation (Ansys FLUENT^®^, v6.3.26). The hydrogel region was approximated as a porous zone with a porosity of 𝜀 = 0.99 and isotropic viscous resistances of *R* = 6.67 ^∗^ 10^−12^ 1/m^2^. Simulations were performed using an oxygen diffusion coefficient of 2 × 10^−9^ m^2^/s.

## Author Contributions

HZ, MR, SS, BB, and PE conceived the project and designed the experimental outline. HZ, MR, SS, and BB performed the microfluidic experiments and analyzed the data. CJ and MH conceived and performed finite volume CFD simulations. BM, JE, and TM prepared the optical sensors for oxygen biosensing. EP, SM, WH, and HR isolated and transfected the primary endothelial and adipose-derived stem cell cultures and shared their expertise in vascular tissue engineering. HZ, MR, SS, and BB drafted the manuscript. All authors contributed to and revised the final manuscript.

## Conflict of Interest Statement

The authors declare that the research was conducted in the absence of any commercial or financial relationships that could be construed as a potential conflict of interest.

## Footnotes

1 www.pyro-science.com

## Supplementary Material

FIGURE S1 Images of microfluidic devices. **(A)** Microfluidic glass chip (76 mm × 26 mm × 2.3 mm) for monolayer culture containing eight chambers with a sensor spot centralized in each chamber. **(B)** Microfluidic glass chip (76 mm × 26 mm × 4.4 mm) for three-dimensional (3D) hydrogel cultures with four sensor spots in each chamber. Scale bar represent 10 mm.

FIGURE S2 Partial oxygen pressure recorded during 3 h of cell seeding zoomed in to the first 50 min of oxygen monitoring.

FIGURE S3 Finite volume simulation of oxygen distribution in 3D hydrogel after **(A)** 1 h, **(B)** 6 h, and **(C)** 12 h of medium perfusion without cells showing complete saturation of hydrogel with oxygen within 12 h.
