# Supplementary figures and images for: Every Breath You Take: Non-invasive Real-Time Oxygen Biosensing in Two- and Three-Dimensional Microfluidic Cell Models
Source: Front Physiol. 2018 Jul 3;9:815. doi: 10.3389/fphys.2018.00815 (PMC6037982; doi:10.3389/fphys.2018.00815)

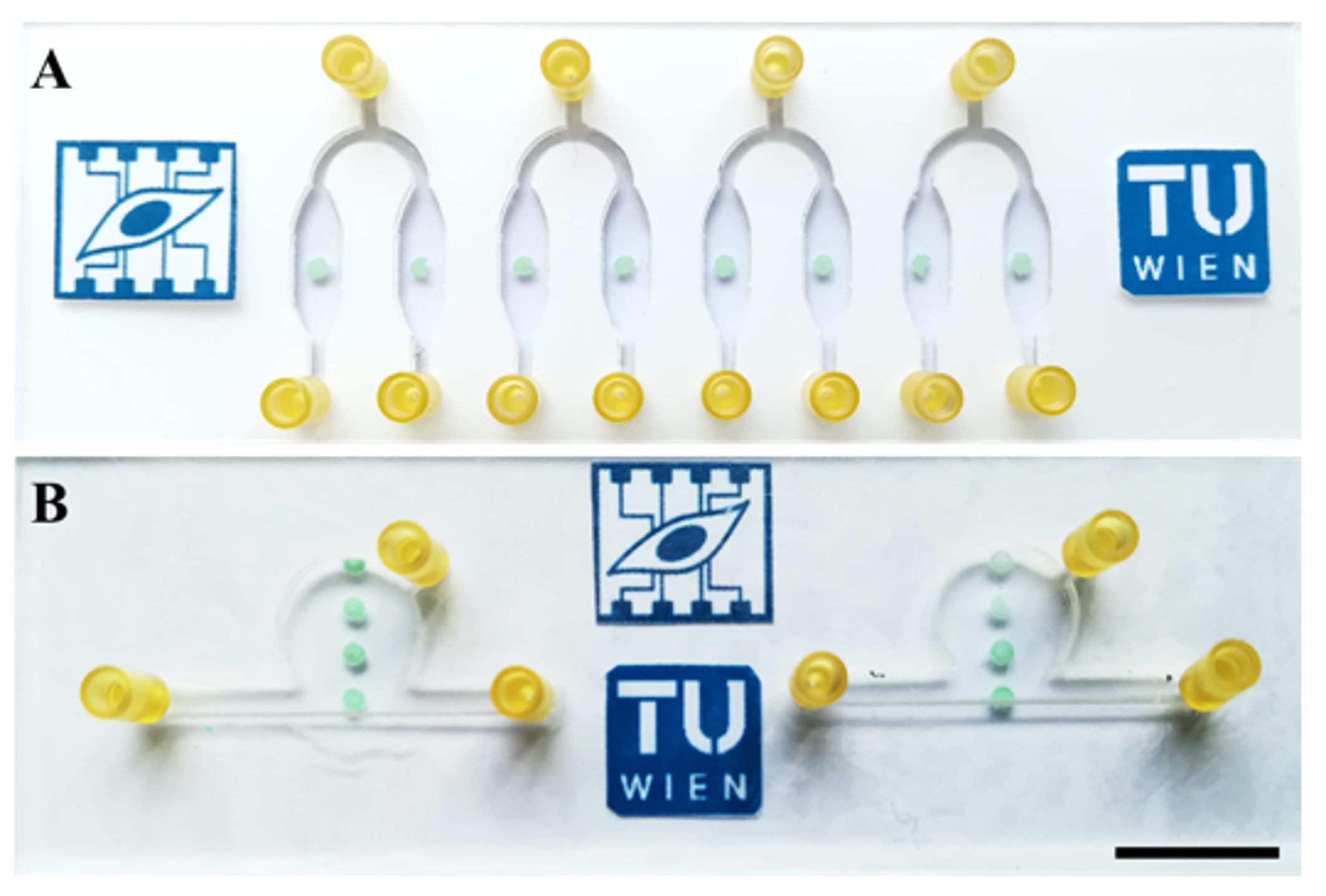

Supplement: FIGURE S1 — Images of microfluidic devices. (A) Microfluidic glass chip (76 mm × 26 mm × 2.3 mm) for monolayer culture containing eight chambers with a sensor spot centralized in each chamber. (B) Microfluidic glass chip (76 mm × 26 mm × 4.4 mm) for three-dimensional (3D) hydrogel cultures with four sensor spots in each chamber. Scale bar represent 10 mm. [file Image_1.TIFF]

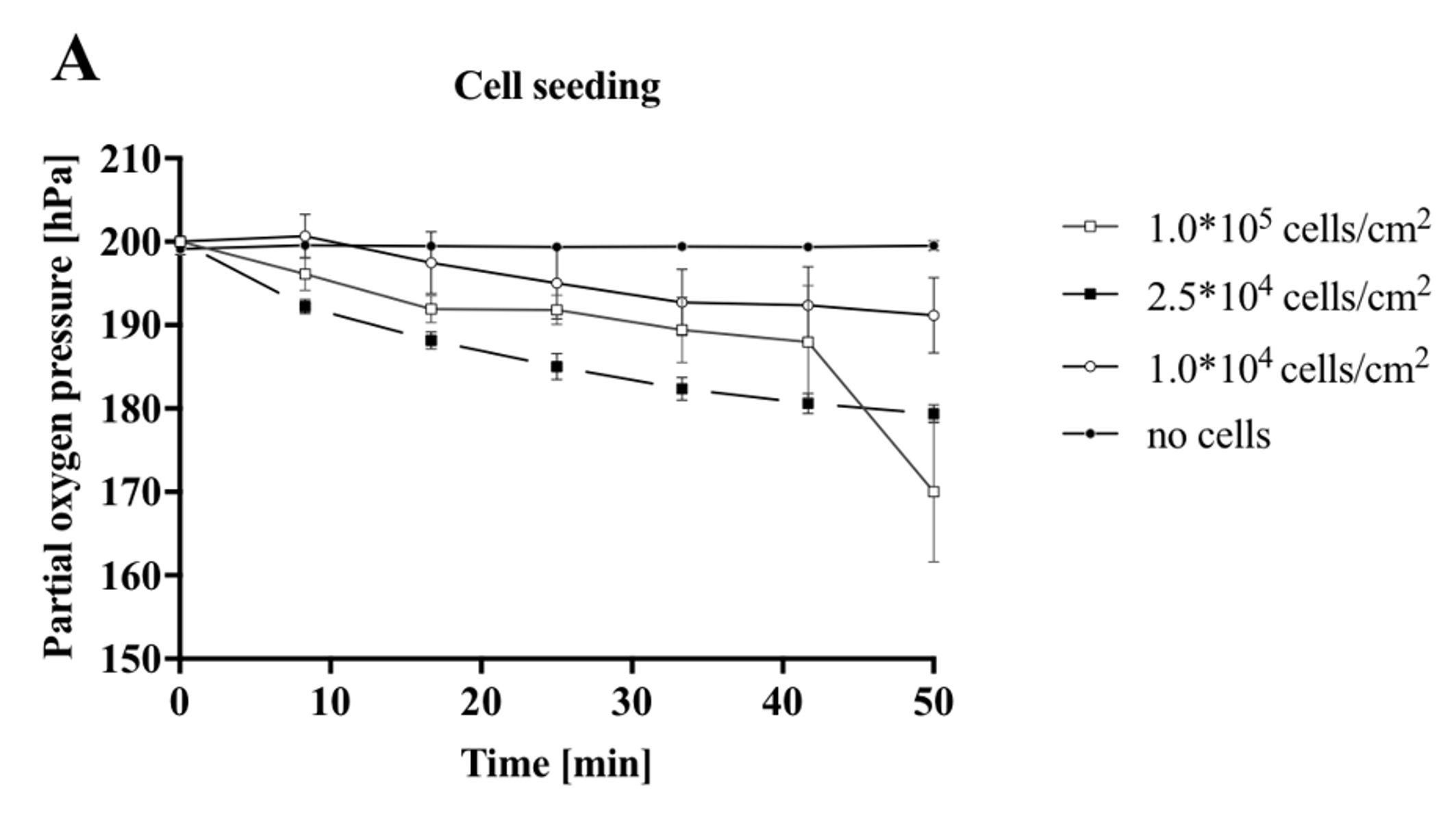

Supplement: FIGURE S2 — Partial oxygen pressure recorded during 3 h of cell seeding zoomed in to the first 50 min of oxygen monitoring. [file Image_2.TIFF]

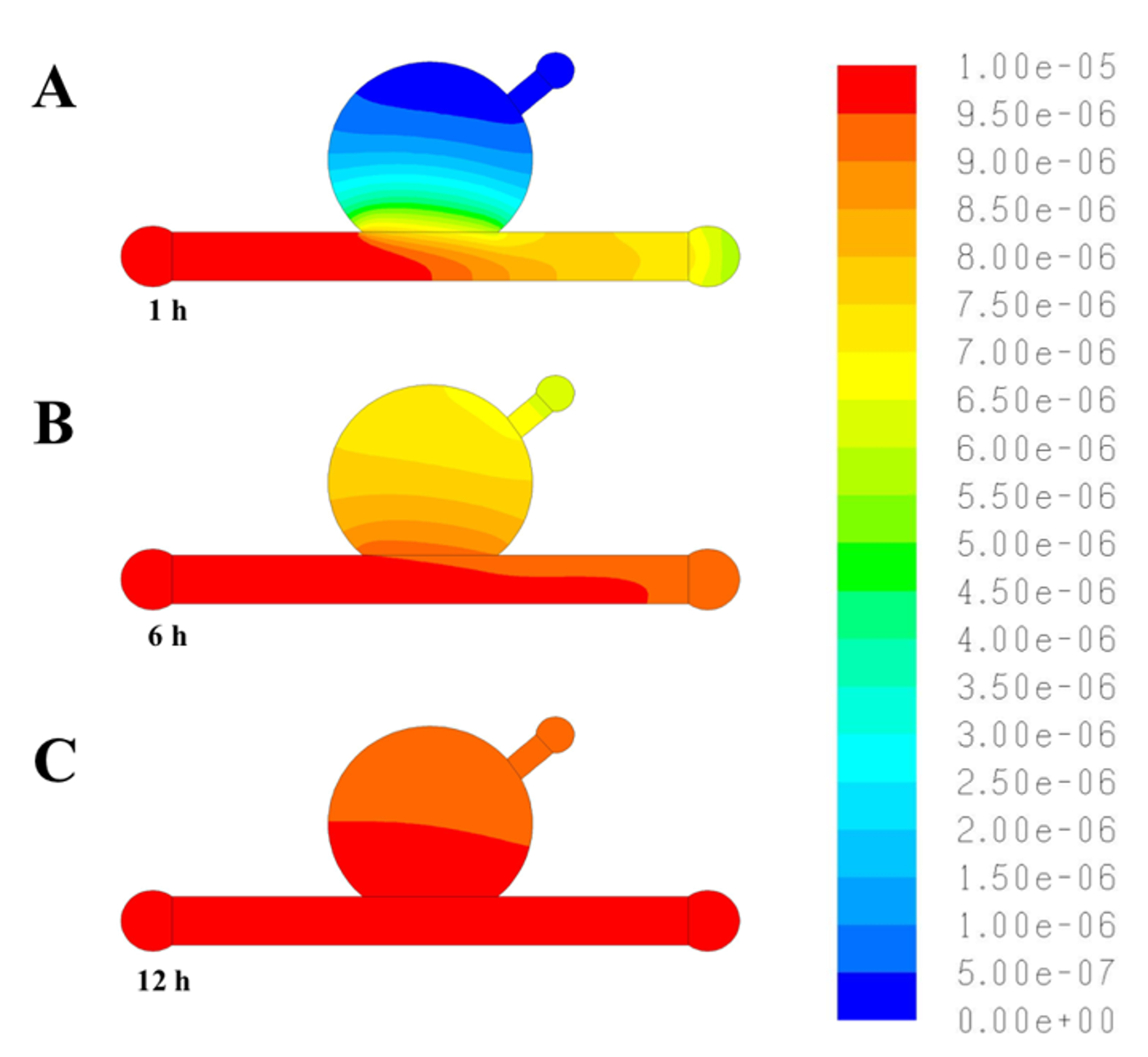

Supplement: FIGURE S3 — Finite volume simulation of oxygen distribution in 3D hydrogel after (A) 1 h, (B) 6 h, and (C) 12 h of medium perfusion without cells showing complete saturation of hydrogel with oxygen within 12 h. [file Image_3.TIFF]
